# Supplementary material for: Pattern transfer of large-scale thin membranes with controllable self-delamination interface for integrated functional systems
Source: Nat Commun. 2021 Nov 26;12:6882. doi: 10.1038/s41467-021-27208-5 (PMC8626417; doi:10.1038/s41467-021-27208-5)
Supplement: Supplementary file 1 — Supplementary Information [file 41467_2021_27208_MOESM1_ESM.pdf]

Supporting Information

Pattern transfer of large-scale thin membranes with controllable self-delamination interface for integrated functional systems

Jun Kyu Park [1] , Yue Zhang [2], Baoxing Xu [2], and Seok Kim[1,3,4]*

[1] Department of Mechanical Science and Engineering, University of Illinois at Urbana-Champaign, Urbana, Illinois 61801, USA
[2] Department of Mechanical and Aerospace Engineering, University of Virginia, Charlottesville, Virginia 22903, USA
[3] Institute for Convergence Research and Education in Advanced Technology, Yonsei University, Seoul, 03722, South Korea
[4] Department of Mechanical Engineering, Pohang University of Science and Technology (POSTECH), Pohang, 37673, South Korea
* To whom correspondence may be addressed. E-mail: seok.kim@postech.ac.kr

Keywords: Pattern transfer, Transfer printing, Self-delamination, Interfacial force

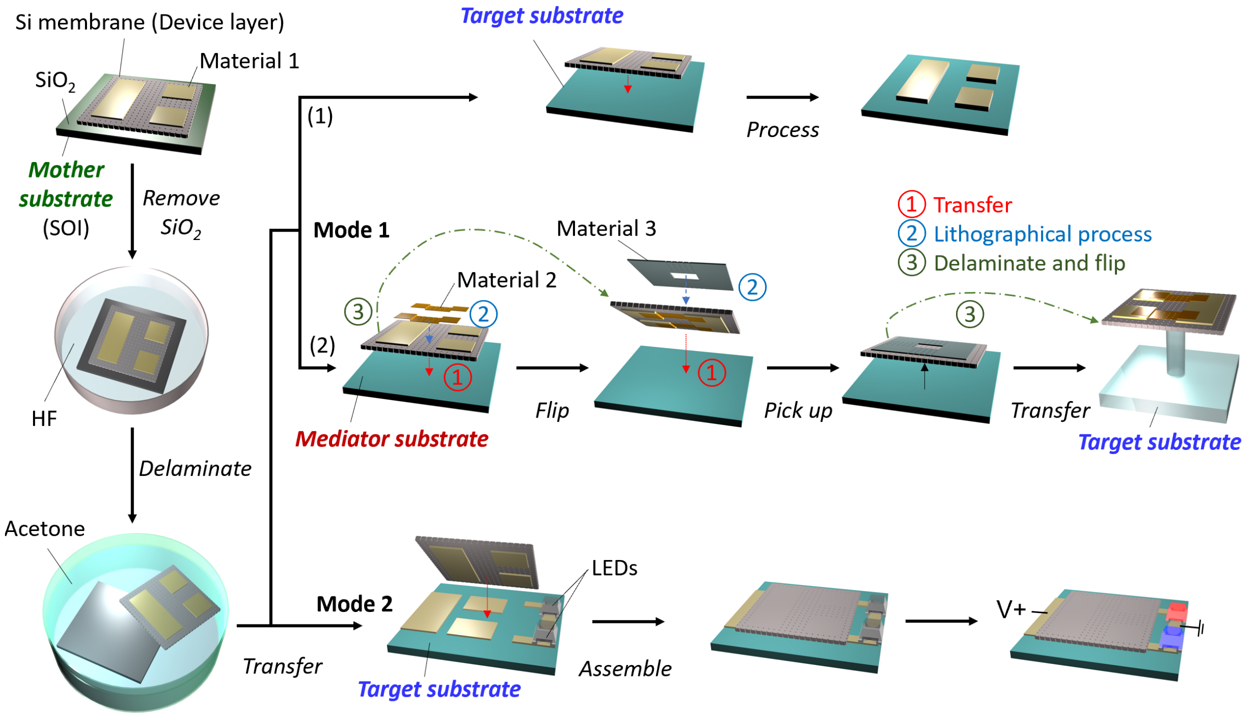

Supplementary Fig. 1 Implementation of the reported pattern transfer with two different modes. Mode I (1st and 2nd rows) starts with the initial patterning of a Si membrane on a mother substrate before transferring either to a target substrate or to a mediator substrate for subsequent processes. If the subsequent processes are finished in a mediator substrate, the patterned Si membrane is finally transferred to a target substrate for use. Mode II (3rd row) involves the initial patterning of a Si membrane on a mother substrate and then transferring to a target substrate without any subsequent process for use.

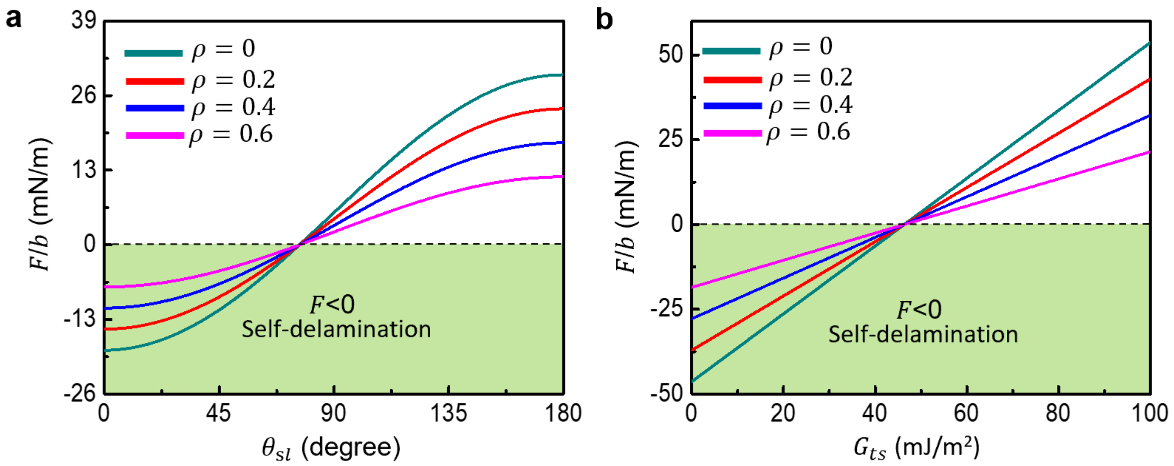

Supplementary Fig. 2 Theoretical analysis on peeling force. **a** Peeling force per unit width F/b as a function of substrate surface wettability θ_{sl} for films with different ρ, where $G_{ts} =$ 29.4mN/m, $\theta_{tl} = 7.65°$, and $\gamma_l = 24$mN/m. **b** Peeling force per unit width F/b as a function of interfacial adhesion energy G_{ts} for films with different ρ, where $\theta_{sl} = 20°$, $\theta_{tl} = 7.65°$, and $\gamma_l = 24$mN/m. $F/b \leq 0$ suggests a self-delamination of film from substrate.

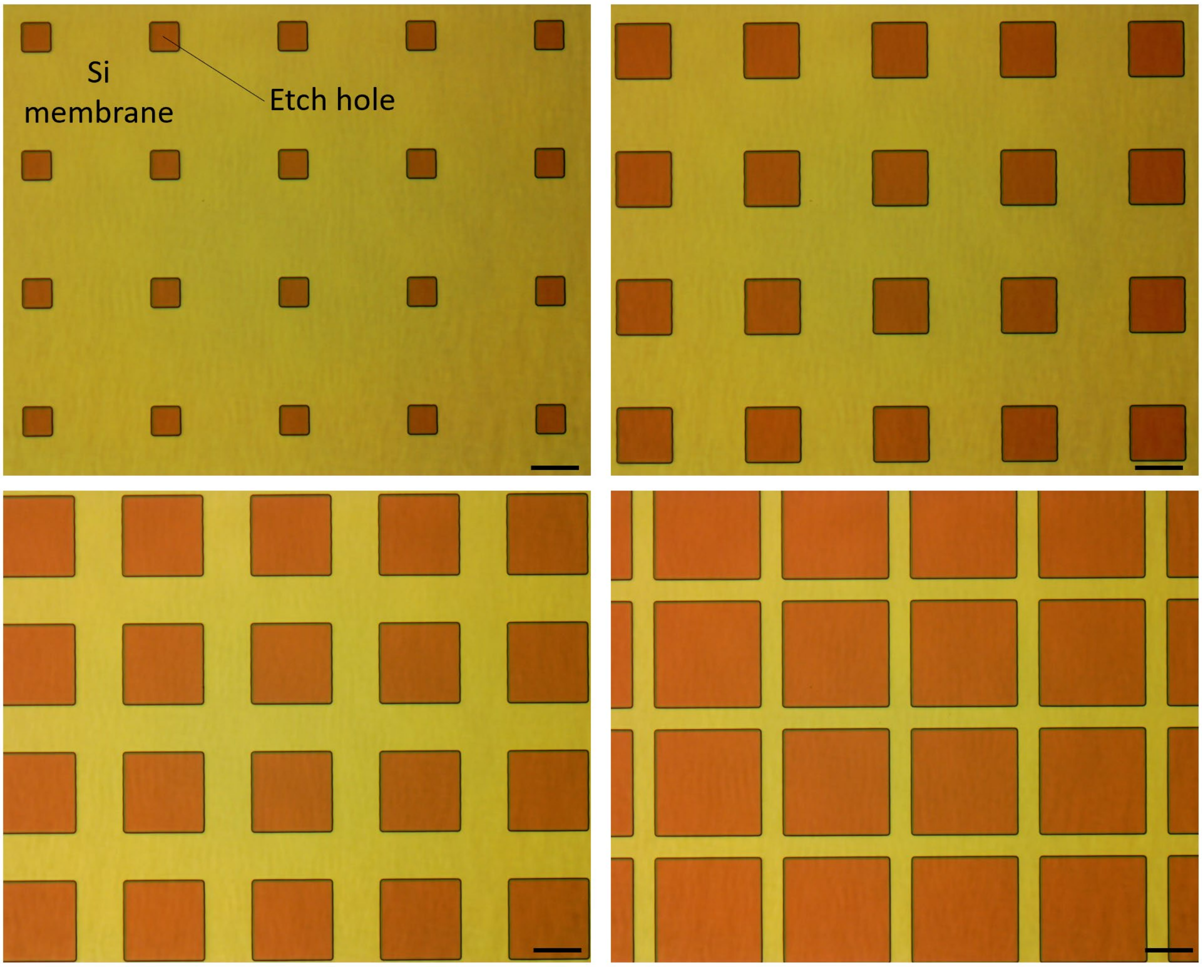

Supplementary Fig. 3 Optical images of Si membranes with different porosity, **a** 0.04, **b** 0.2, **c** 0.4, **d** 0.7. Scale bars indicate 100 μm.

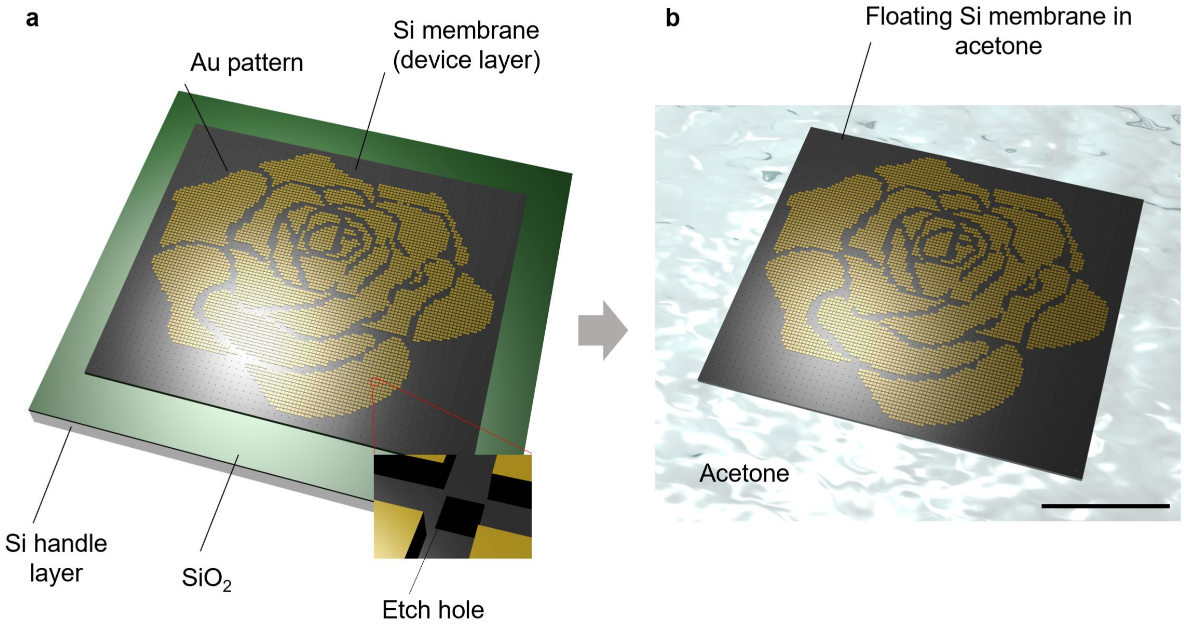

Supplementary Fig. 4 Schematic illustration of initial processing state of Si platelet patterning on a mother substrate. **a** A Si membrane with a Au pattern on SiO_2 layer. Inset image shows an etch hole defined in the Si membrane. **b** Floating Si membrane in acetone bath after removing SiO_2 in HF bath and submerging in acetone bath. Scale bar indicates 5 mm.

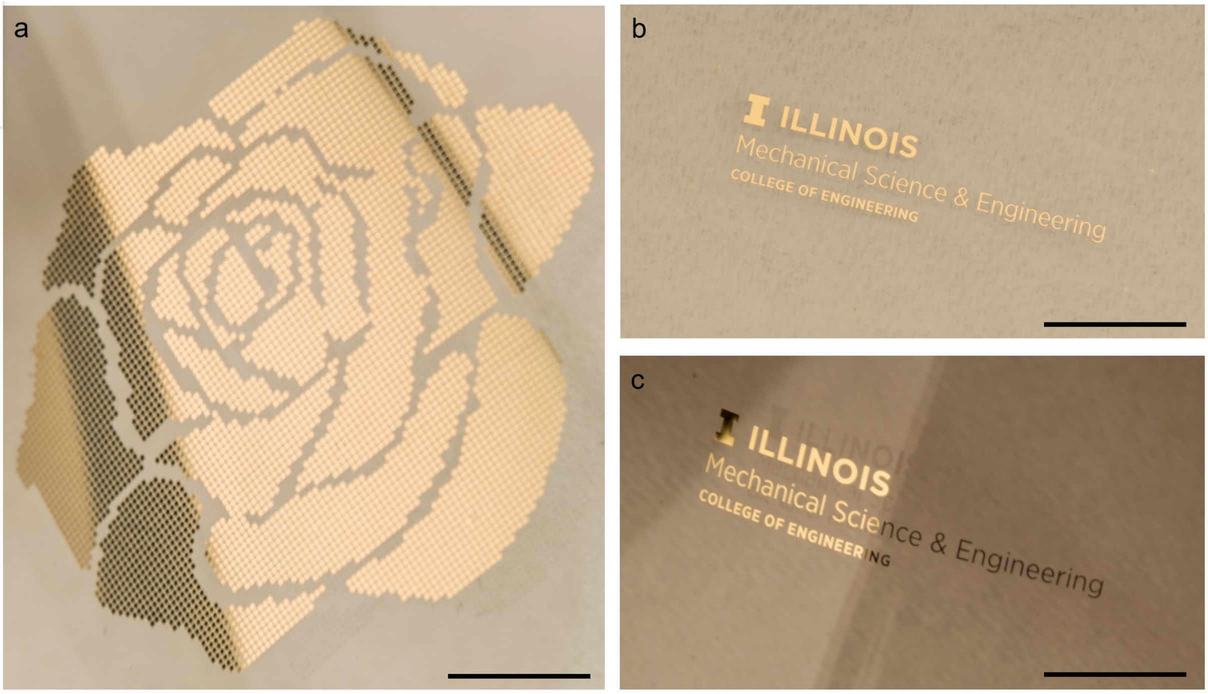

Supplementary Fig. 5 Optical images of Si platelet arrays on glass and curved PDMS substrates. **a** Rose mosaic art on curved PDMS slab **b** University logo on a glass substrate and **c** on a curved PDMS substrate. Scale bars indicate 5 mm.

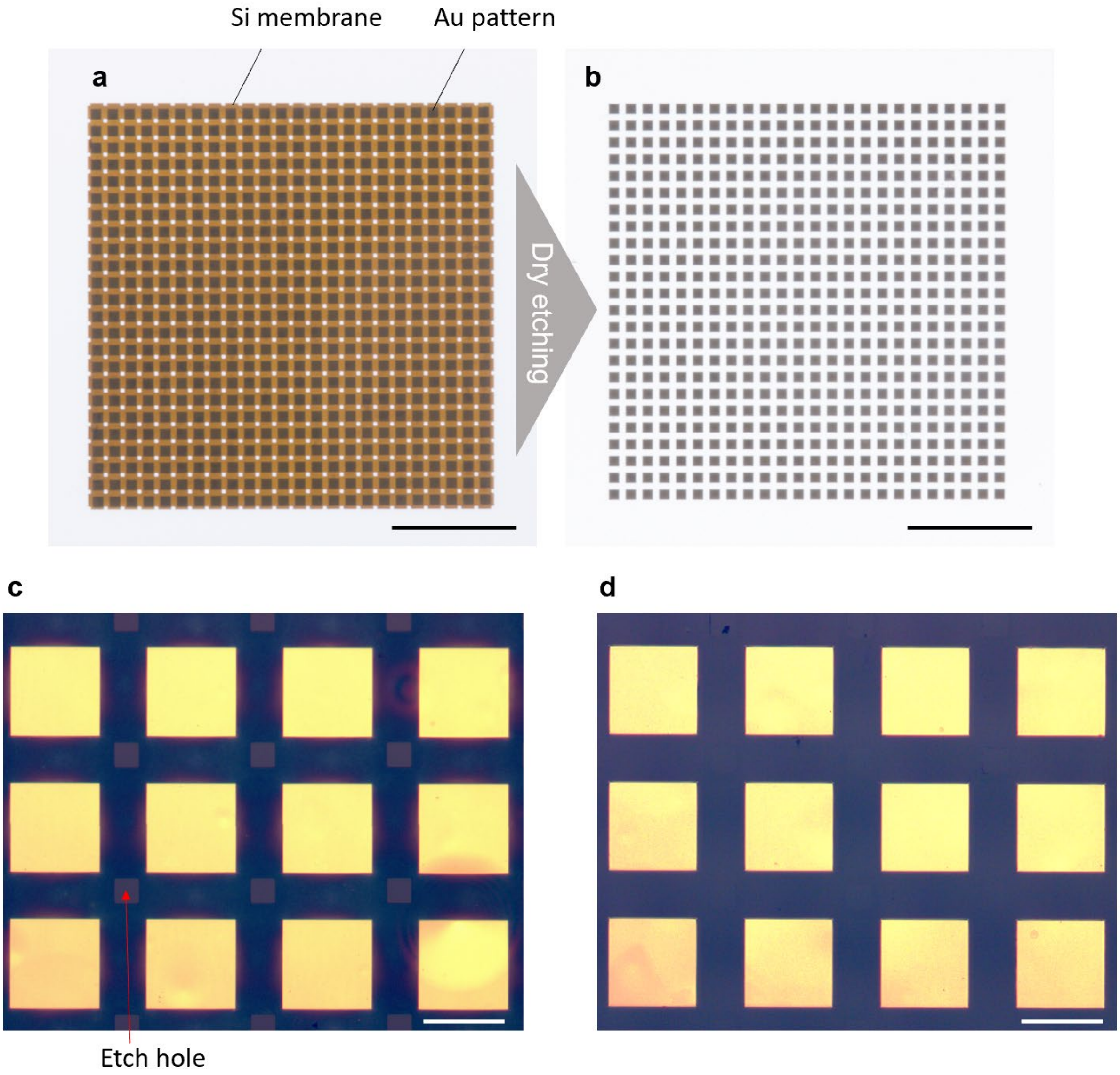

Supplementary Fig. 6 Optical images of Au/Si platelet array processing on a silanized glass substrate. **a** Optical image of a Si membrane with a square Au array. **b** Optical image of dissected Au/Si platelets that are readily transfer printed. Scale bars indicate 1 mm. **c** Magnified image of the Si membrane. **d** Magnified image of the Au/Si platelet array. Scale bar indicates 100 µm.

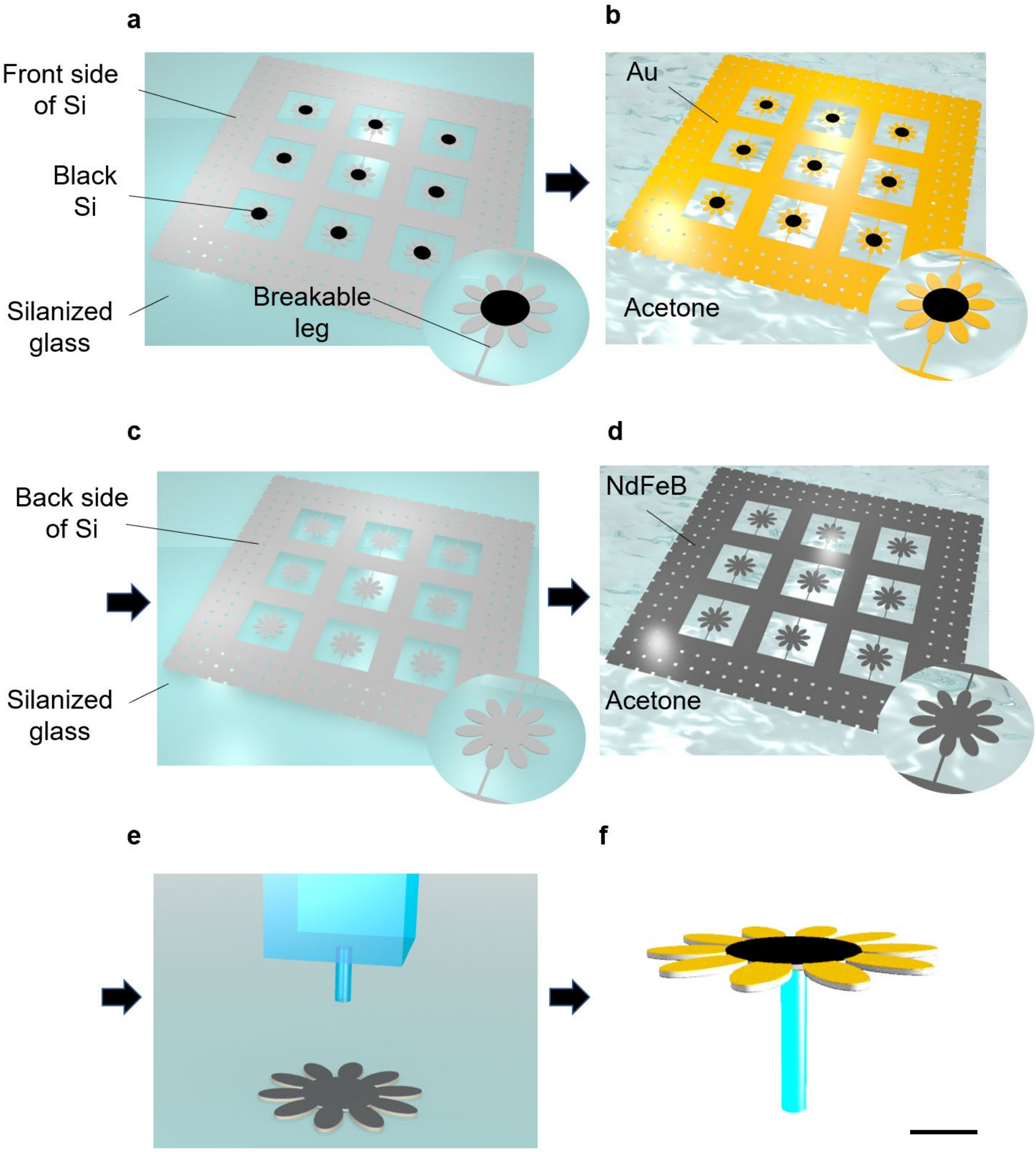

Supplementary Fig. 7 Schematic illustrations of Si platelet double-side patterning. **a** A Si membrane with sunflower-shape black silicon patterned is transferred on a silane coated glass substrate. **b** A Au layer is patterned on the Si membrane which is delaminated and floated in acetone bath. **c** The Si membrane is film-transferred on a new silanized glass substrate for backside processing. **d** A NdFeB magnetic alloy is sputtered on the back side and the Si membrane is delaminated from the substrate and is floated in acetone bath. **e** The Si membrane is transferred on another new silanized glass substrate. After magnetization, one Si platelet that is mechanically tethered from the Si membrane is picked up by a PDMS pillar. **f** The fabricated sunflower mimicking structure. Scale bars indicate 300 μm.

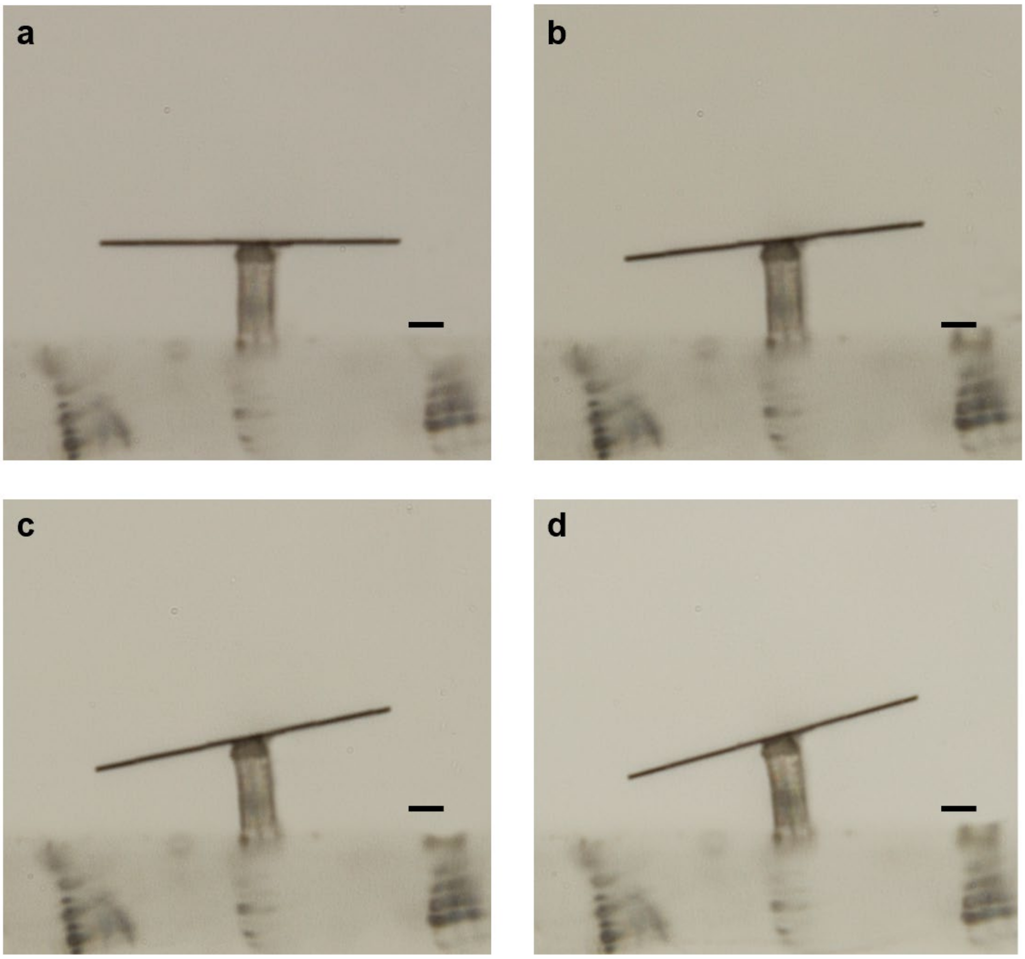

Supplementary Fig. 8 Optical images of sunflower mimicking structure under different magnetic field. **a** 0 T **b** 0.55 T **c** 0.7 T **d** 0.8 T. Scale bars indicate 100 μm.

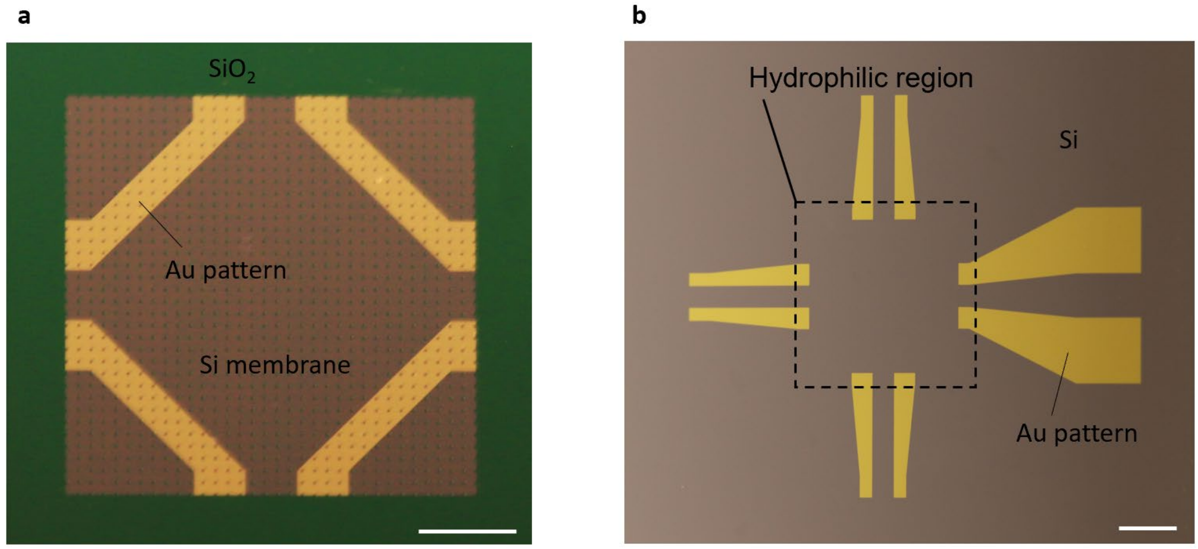

Supplementary Fig. 9 Optical images of assembly parts of LED circuit. **a** A Si membrane with disconnected Au wire pattern. **b** A target substrate with counter Au wire pattern with square hydrophilic region. Scale bars indicate 2 mm.

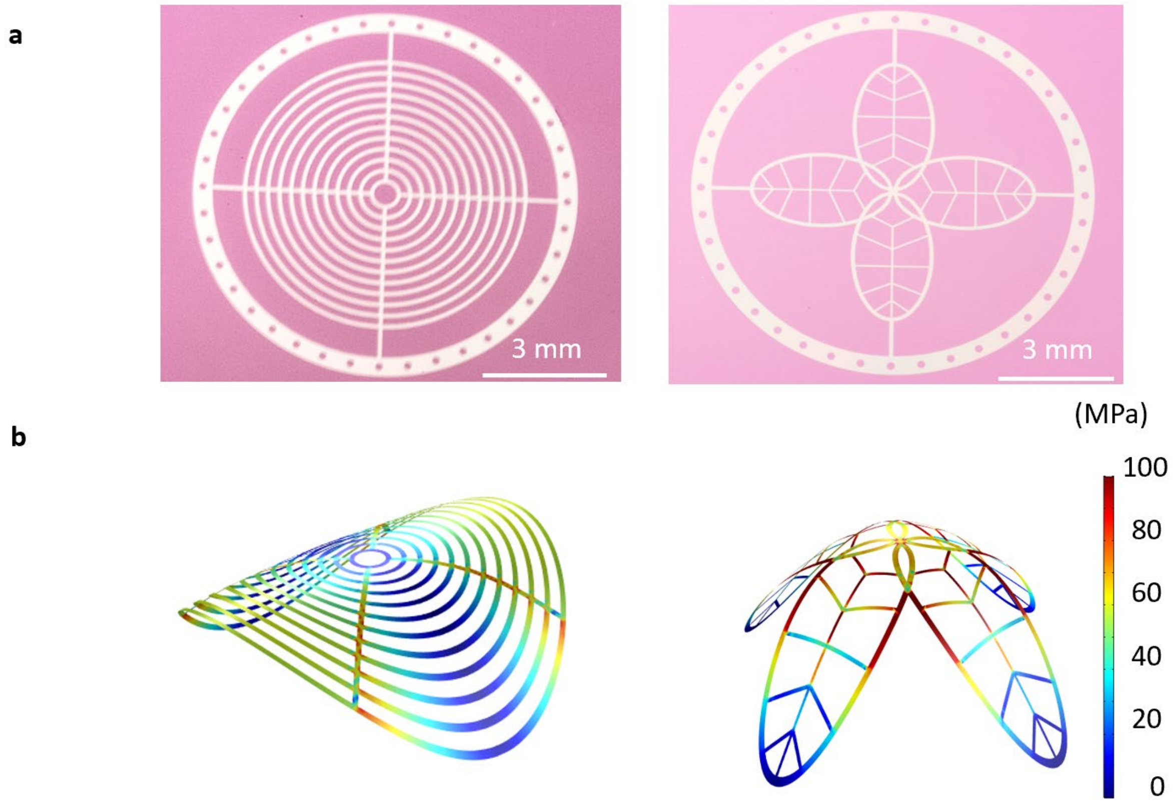

Supplementary Fig. 10 Patterned Si membranes for single crystalline Si 3D mesostructures. **a** Optical images of two different Si membrane design. **b** FEA contour plots that show first principal stress and predict the shape of 3D mesostructures for each case. The first principal stress does not exceed failure strength of single crystalline Si.

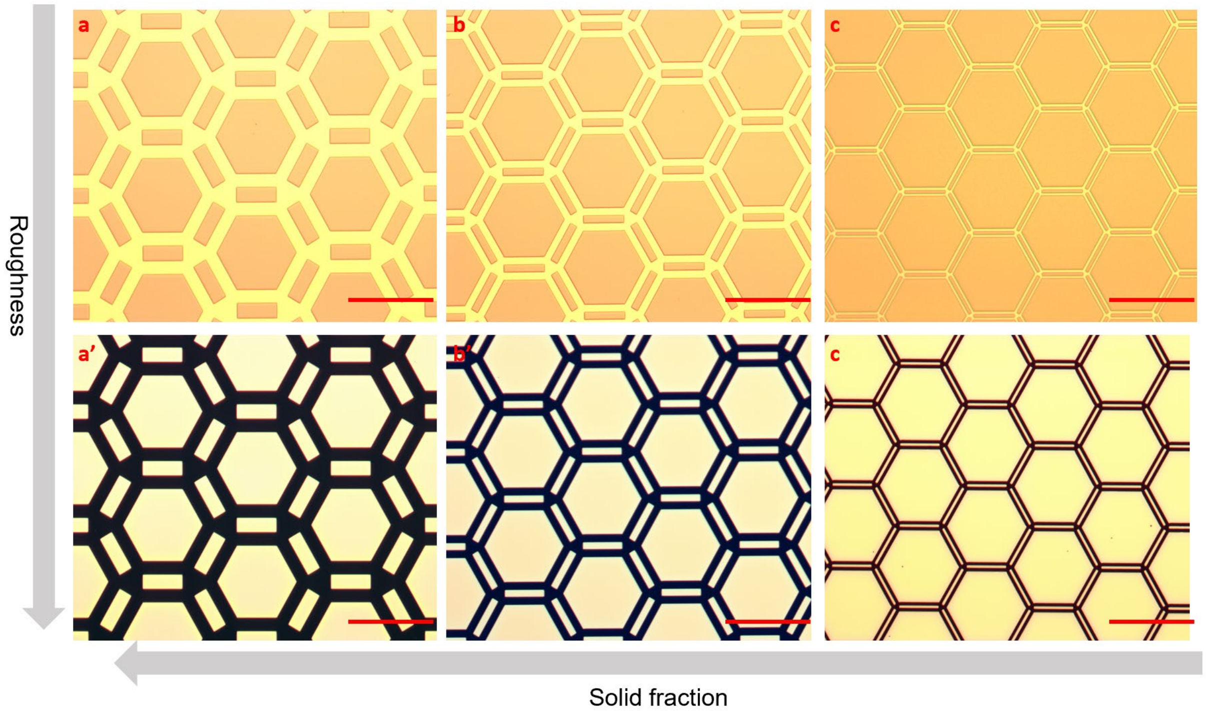

Supplementary Fig. 11 Top view optical images of porous Si membranes with different designs. **a** Si membrane with high solid fraction, f=0.44 **b** Si membrane with medium solid fraction, f=0.3 **c** Si membrane with low solid fraction, f=0.13. Lower images, **a'**, **b'**, and **c'** indicates nanostructured (r_f = 2.8) Si membranes with corresponding solid fractions, respectively. Scare bars indicate 200 µm.

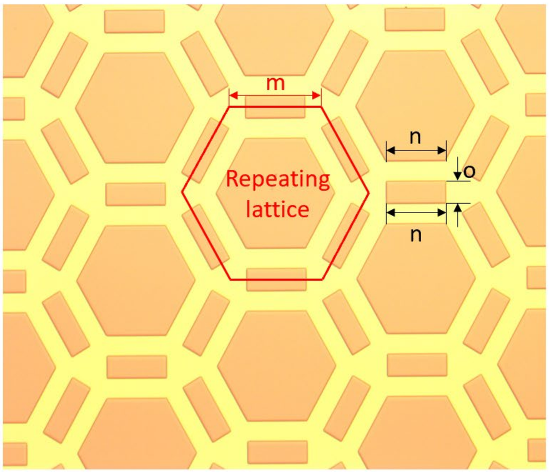

	Si/SU8			Si/PDMS		
	a	b	c	a	b	c
m	193	164	138	193	164	138
n	125	125	125	125	125	125
o	N/A	N/A	N/A	37	22	7
f	0.58	0.42	0.18	0.44	0.3	0.13
	$1-\left(\frac{n}{m}\right)^2$			$1-\left(\frac{n}{m}\right)^2-\frac{2\sqrt{3}\cdot n\cdot o}{3\cdot m^2}$		

Supplementary Fig. 12 Method to calculate solid fraction of Si/SU8 and Si/PDMS surfaces based on geometric parameters of three different Si membrane designs labeled 'a', 'b', 'c'.

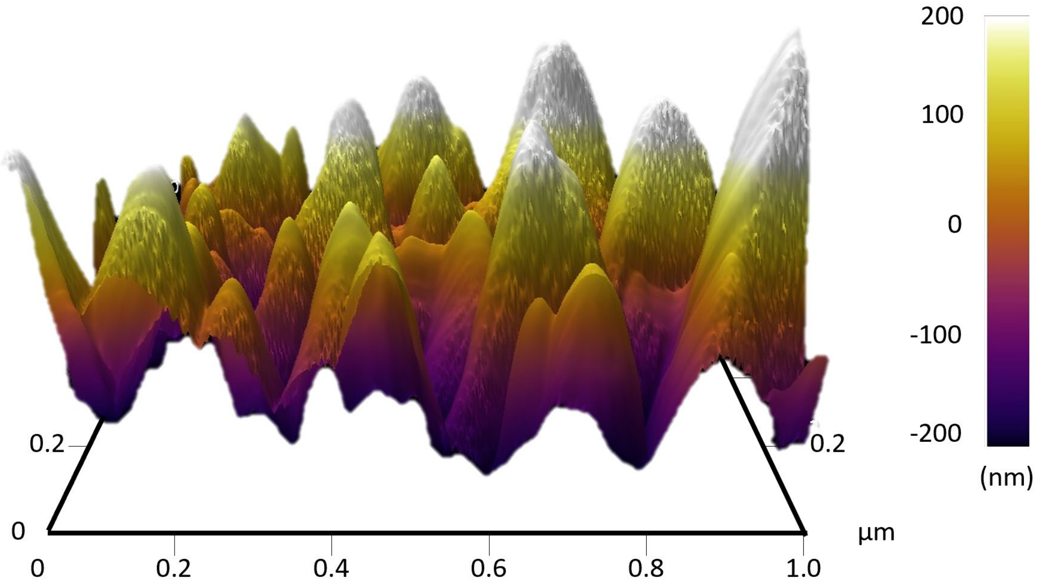

Supplementary Fig. 13 AFM image over 1µm by 1µm area of black silicon surface. The scale bar indicates that the roughness ratio r_f is 2.8 on average.

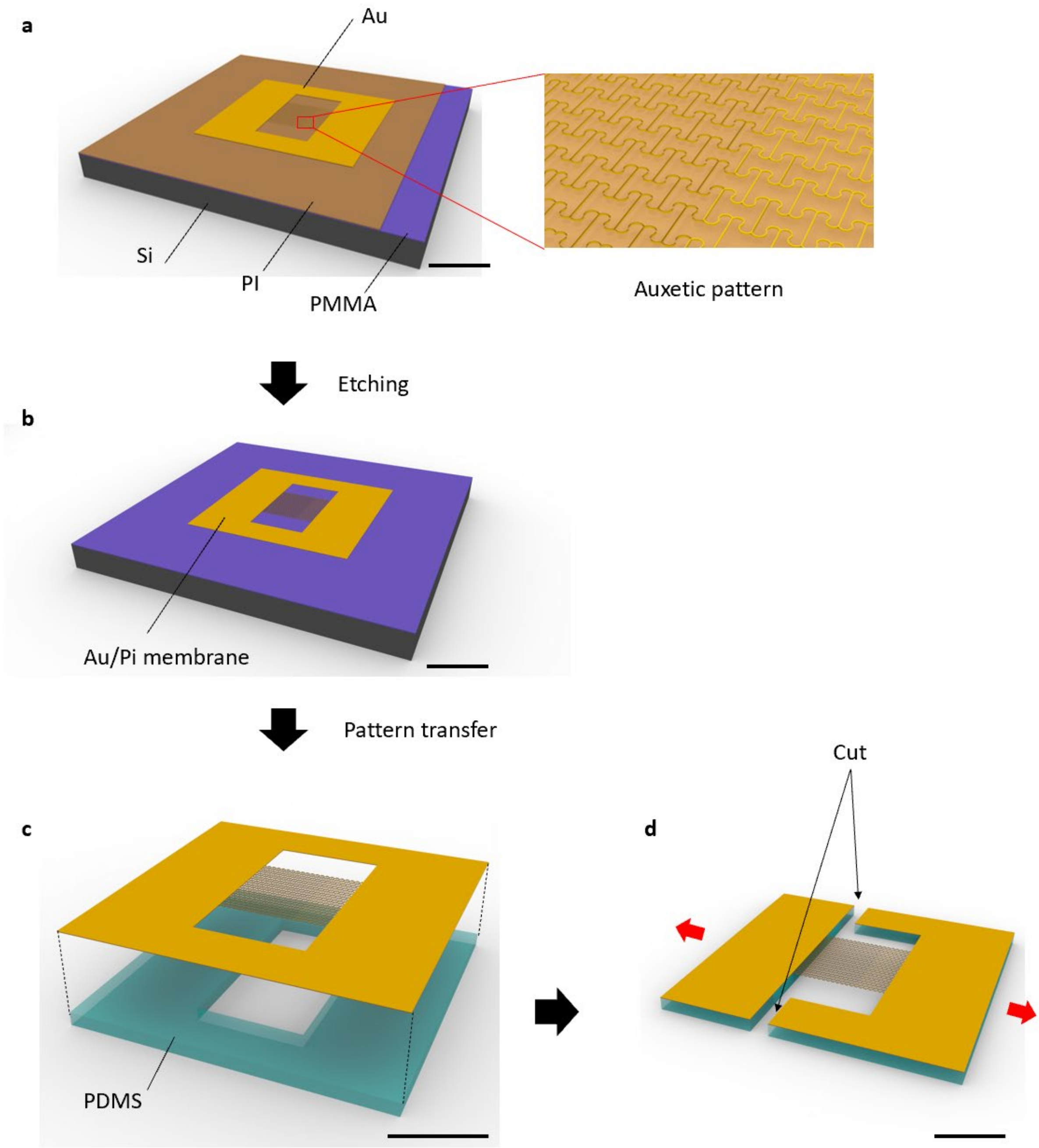

Supplementary Fig. 14 Schematic illustrations of the fabrication of stretchable auxetic membranes. **a** Pattern Au on PI and PMMA coated Si substrate. **b** Etch PI using Au as an etching mask. **c** Delaminate Au/PI membrane from Si substrate by dipping into acetone bath and transfer it onto shaped PDMS. **d** Uniaxially stretch auxetic membrane after mechanically cutting edges.

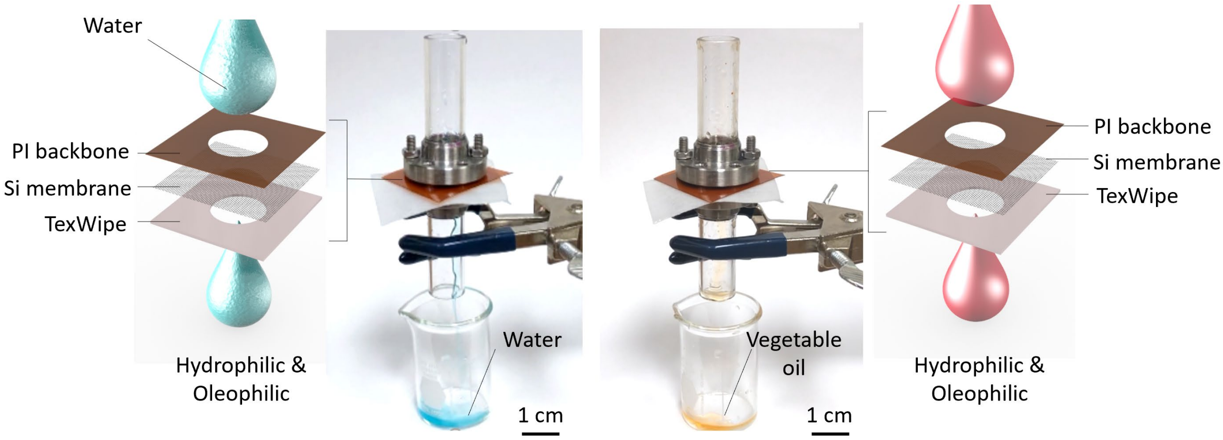

Supplementary Fig. 15 Application to omniphilicity. Assembled PI/Si/TexWipe® shows omniphilicity. Water as well as vegetable oil pass through the assembled surface.

Supplemetary Table 1 The values of *G*, contact angle, and surface tension for various combination of film, substrate, and liquid.

Si/Si (HF treated) with water	G_{ts} = 47.3mN/m , γ_l = 72mN/m and θ_{tl}=θ_{sl}= 78.5°
Si/Si (HF treated)with acetone	G_{ts} = 47.3mN/m, γ_l = 24mN/m and θ_{tl}=θ_{sl}= 7.65°
(HF treated) Si/Glass with acetone	G_{ts} = 70.2mN/m, γ_l = 24mN/m and θ_{tl}=5.75° θ_{sl}= 5.75°
(HF treated) Si/Glass with water	G_{ts} = 70.2mN/m, γ_l = 72mN/m and θ_{tl}=78.85° θ_{sl}= 24.66°
(HF treated) Si/silanized Glass with acetone	G_{ts} = 29.4mN/m, γ_l = 24mN/m, and θ_{tl}=7.56° θ_{sl}= 20°
(HF treated) Si/silanized Glass with water	G_{ts} = 29.4mN/m, γ_l = 72mN/m, and θ_{tl}=78.85° θ_{sl}= 107.72°
