# Supplementary material for: Pattern transfer of large-scale thin membranes with controllable self-delamination interface for integrated functional systems
Source: Nat Commun. 2021 Nov 26;12:6882. doi: 10.1038/s41467-021-27208-5 (PMC8626417; doi:10.1038/s41467-021-27208-5)
Supplement: Supplementary file 3 — Description of Additional Supplementary Files [file 41467_2021_27208_MOESM3_ESM.pdf]

**Description of Additional Supplementary Files**

File Name: Supplementary Movie 1
Description: It shows the self-alignment procedure for the LED circuit.

File Name: Supplementary Movie 2
Description: It shows the detailed experimental results related to Fig. 7a.

File Name: Supplementary Movie 3
Description: It shows the directional omniphobicity of the membrane.

File Name: Supplementary Movie 4
Description: It shows the separation of chloroform and water using the membrane.

File Name: Supplementary Movie 5
Description: It shows the directional omniphilicity of the membrane.
